# Editorial: Targeted and untargeted metabolomics for the evaluation of plant metabolites in response to the environment

**DOI:** 10.3389/fpls.2023.1167513

**Published:** 2023-03-03

**Authors:** Wenyan Han, Jane L. Ward, Yingzhen Kong, Xin Li

**Affiliations:** ^1^ Key Laboratory of Tea Quality and Safety Control, Ministry of Agriculture and Rural Affairs, Tea Research Institute, Chinese Academy of Agricultural Sciences, Hangzhou, China; ^2^ Plant Sciences and the Bioeconomy, Rothamsted Research, Harpenden, United Kingdom; ^3^ College of Agronomy, Qingdao Agricultural University, Qingdao, China

**Keywords:** metabolomics, mass spectrometry, gas chromatography, liquid chromatography, metabolic profiling, crop quality, stress response

Over 200 thousand unique metabolites constitute the extremely intricate and diverse plant metabolome (Sousa Silva et al., 2019). Understanding plant growth, development, and responses to environmental perturbations largely rely on the identification and characterization of these diverse metabolites (Allwood et al., 2021). Metabolomics allows the comprehensive study of the complex biochemistry of plant-environment interactions, enabling researchers to better understand plant growth as well as stress responses (Wu et al., 2023). Thus, applications of metabolomics to plant science hold tremendous promise. Metabolomics can broadly be classified into un-targeted and targeted metabolomics (Allwood et al., 2021). Untargeted metabolomics attempts to analyze all detectable metabolites from the sample, including unknown compounds (Christ et al., 2018). However, targeted metabolomics focuses on the analysis of specific categories of metabolites with more selectivity and sensitivity (Roberts et al., 2012). Nonetheless, both un-targeted and targeted metabolomics have benefits and drawbacks, and they are often employed together to detect and precisely quantify diverse metabolites (Allwood et al., 2021; Wu et al., 2023). Currently, multiple analytical platforms allow both targeted metabolite profiling and untargeted metabolic fingerprinting using different strategies. This Research Topic is a compilation of 5 research articles that highlight the state-of-the-art and cutting-edge methods of metabolomics in plant research.

Light, one of the most important environmental factors for plants, not only drives photosynthesis but also alters the nutritional contents and compositions of edible plant products (Xu et al., 2021; Ahammed et al., 2022). Grape (*Vitis vinifera* L.) berry seeds are rich in oil content and have nutraceutical and pharmacological values. Interestingly, grape seeds are capable to continue photosynthetic activity even at the mature stage (Garrido et al., 2021). Garrido et al. followed an untargeted metabolomics approach using liquid chromatography coupled with high-resolution mass spectrometry (LC-MS) to analyze the lipid profiles of grape seeds at different stages of berry development under shaded and fully exposed light conditions. Compared with light conditions, developmental stages greatly influenced the lipid profiles of grape seeds. Notably, the shaded conditions triggered the accumulation of seed fatty acids at the mature stage which potentially contributed to the synthesis of storage lipids. In contrast, light exposure increased the abundance of ceramides at the green stage and upregulated the levels of triacylglycerols and glycerophospholipids at the mature stage, which might contribute to photosynthetic activity in mature seeds. These findings point to the possibility that photosynthesis in grape berry seeds serves different functions at different stages of development, perhaps by providing energy to different lipid pathways. Light exposure also affects the biomass and quality of alfalfa (*Medicago sativa* L.) sprouts, one of the most nutritionally rich foods (Zhang et al., 2020a). Zhang et al. integrated metabolomics and transcriptomics approaches to assess the composition of nutrients in germinating alfalfa sprouts in the presence or absence of light. They conducted targeted metabolomics of yellow and green alfalfa sprouts using ultra-performance liquid chromatography (UPLC) coupled with a tandem mass spectrometry (MS/MS) system. Among a range of lipids, phenols, flavonoids, and terpenoids in green alfalfa sprouts, the three most abundant metabolites were calycosin, methyl gallate, and epicatechin 3-gallate, while isoquercitrin was particularly abundant in yellow alfalfa sprouts. The study provides valuable information on the nutritional content and economic value of alfalfa sprouts under different light environments and supports the efficacy of targeted metabolomics for the comprehensive study of the sprout metabolic profiles as a reference for future research.

Polyamines such as spermine are important signaling molecules that play a crucial role in plant response to abiotic stress (Zhang et al., 2020b). Li et al. studied the effects of exogenous spermine on the reprogramming of global metabolites and their related metabolic pathways in creeping bentgrass (*Agrostis stolonifera*) as influenced by drought and/or heat stress. They used gas chromatography (GC)-time-of-flight mass spectrometry (TOFMS) to measure the leaf metabolome and found 61 metabolites that were differentially regulated by spermine, including increased abundances of mannose, galactose, maltose, and urea, associated with improved osmotic adjustment and maintenance of growth under stress conditions. This work presents unique evidence of how spermine-induced altered global metabolites contribute to growth maintenance and responses to drought and heat stress. Tea (*Camellia sinensis* L.), one of the most popular beverage crops in the world prefers humid environments and thus water deficit largely affects tea yield and quality (Han et al., 2018). Shen et al. used targeted metabolomics and lipidomics approaches with GC-MS analysis and HPLC-MS/MS to analyze the variations in the accumulation of leaf metabolites and lipids in response to drought and re-watering. Drought stress substantially altered 119 metabolites, including various sugars, amino acids, and lipids in tea leaves. The study provides convincing evidence that integrated metabolomics and lipidomics techniques are effective in revealing the effects of drought stress on tea plant metabolism, and better understanding the processes underlying drought tolerance in tea plants.

*Stellaria dichotoma* L. var. lanceolata Bge (SDL), a Chinese medicinal plant rich in active sterols and flavonoids is found mostly in semi-arid and arid regions of China as the primary habitat (Chinese Pharmacopoeia Commission, 2020). To assess the effect of origin and habitats on SDL metabolites and quality, Li et al. used ultra-HPLC-quadrupole-TOF MS-based metabolomics to analyze the metabolites of SDL from nine different production areas. They identified 1586 distinct metabolites from different habitats, spanning 13 classes including organic acids and lipids. The study concluded that diverse habitat factors have different effects on SDL metabolites and quality, which may guide the selection of appropriate habitat factors for the efficient production of this plant species with rich target metabolites.

Understanding how changes in plant metabolites affect crop quality and stress tolerance is essential to developing innovative approaches aiming to improvement of crop yield and quality in the face of climate change. This Research Topic is a timely collection of advanced metabolomics studies. However, further study is needed to better understand the regulation mechanism of plant growth, development, and stress responses. We hope that this Research Topic serves as a starting point for future discussion of the many applications of metabolomics to the study of plants.

## Author contributions

WH and XL wrote the article. All authors revised and approved the article.
